# Systemic Lupus Erythematosus before and after COVID-19 Lockdown: How the Perception of Disease Changes through the Lenses of Narrative Medicine

**DOI:** 10.3390/healthcare9060726

**Published:** 2021-06-12

**Authors:** Fulvia Ceccarelli, Venusia Covelli, Giulio Olivieri, Francesco Natalucci, Fabrizio Conti

**Affiliations:** 1 Lupus Clinic, Reumatologia, Dipartimento di Scienze Cliniche, Internistiche, Anestesiologiche e Cardiovascolari, Sapienza Università di Roma, 00161 Roma, Italy; giulio.olivieri@uniroma1.it (G.O.); francesco.natalucci@uniroma1.it (F.N.); fabrizio.conti@uniroma1.it (F.C.); 2 Faculty of Psychology, eCampus University, Novedrate, 22060 Como, Italy; venusia.covelli@uniecampus.it

**Keywords:** Systemic Lupus Erythematosus, COVID-19, narrative medicine, patient’s perspective

## Abstract

Background: The COVID-19 pandemic contributes to the burden of living with different diseases, including Systemic Lupus Erythematosus (SLE). We described, from a narrative point of view, the experiences and perspectives of Italian SLE adults during the COVID-19 emergency, by distinguishing the illness experience before and after the lockdown. Methods: Fifteen patients were invited to participate. Illness narratives were collected between 22 and 29 March 2020 using a written modality to capture patients’ perspectives before and after the COVID-19 lockdown. We performed a two-fold analysis of collected data by distinguishing three narrative types and a qualitative analysis of content to identify the relevant themes and sub-themes reported. Results: Eight narratives included in the final analysis (mean length 436.9 words) have been written by eight females (mean age 43.3 ± 9.9 years, mean disease duration 13.1 ± 7.4 years). Six patients provided a quest narrative, one a chaos and the remaining one a restitution narrative. By text content analysis, we identified specific themes, temporally distinct before and after the lockdown. Before COVID-19, all the patients referred to a good control of disease, however the unexpected arrival of the COVID-19 emergency broke a balance, and patients perceived the loss of health status control, with anxiety and stress. Conclusions: We provided unique insight into the experiences of people with SLE at the time of COVID-19, underlining the perspective of patients in relation to the pandemic.

## 1. Introduction

COVID-19, an infectious disease caused by the SARS-CoV-2 virus, has been classified as a pandemic by the World Health Organization (WHO) [1]. The emergency due to COVID-19 could significantly influence the burden of life of patients affected by different pathological conditions, including Systemic Lupus Erythematosus (SLE).

SLE is an autoimmune condition involving different organs and systems. Its pathogenesis is not fully understood, but evidence have demonstrated a multifactorial etiology, including genetic, environmental and hormonal factors [2,3]. Several comorbidities could affect SLE patients by increasing their mortality, including cardiovascular disease and infections [4,5].

In particular, infections represent one of the most important comorbidities in SLE patients and, in this regard, treatment with immunosuppressant drugs could further increase such risk [5]. Thus, the presence of a pandemic certainly represents a source of concern in SLE patients. Nonetheless, the use of hydroxychloroquine (HCQ) in COVID-19 treatment determined a drug shortage, leading to drug reduction or stopping in the first lockdown [6,7]. Thus, COVID-19 infection, and the derived lockdown, have certainly contributed to the burden of living with SLE.

In the present analysis, we aimed at describing the experiences and perspectives of Italian adults living with SLE during the COVID-19 emergency. In a temporal sense, we investigated the illness experience, distinguishing before and after the COVID-19 lockdown.

## 2. Materials and Methods

We invited to participate fifteen SLE patients, diagnosed according to 1997 ACR criteria and attending at the Lupus Clinic of Sapienza University of Rome [8]. The participation in the study was on a voluntary basis and each patient provided written consent to participation.

Demographic data (gender, age, marital status, level of education, work condition), clinical features, laboratory parameters and treatments were collected from patients’ clinical records. Moreover, we registered disease activity by using the SLEDAI-2k [9].

Patients’ illness narratives were collected between 22 and 29 March 2020 using a written modality. We chose this modality due to the intention of analyzing their illness narratives spontaneously written and without interruptions by the interviewer. As the goal of the present study was to describe the impact of the COVID-19 pandemic on the illness experience in a temporal sense, we provided a semi-structured interview which included four questions, as reported below:

1. How did you consider your illness before the COVID-19 emergency?
2. How did you consider your illness after the COVID-19 emergency?
3. What do you think about your future in light of the COVID-19 emergency?
4. Would you like to write other comments, ideas, or impressions related to your COVID-19 experience?

### Data Analysis

A two-fold analysis of collected data was performed. According to narrative methods described by Greenhalgh et al. in 2005, we analyzed the stories as a whole without segmentation of the text [10] and we applied the classification proposed by Frank in 1998, by distinguishing three narrative types: restitution narrative, chaos narrative and quest narrative [11].

The restitution narrative focuses on the possible restoration of old identity and it is related to the concept of disease, in which the treatment is able to regain a condition of normality. The chaos narrative is generally poorly structured and orderly, and it can be difficult to understand due to the lack of a timeline. This narrative indicates the lack of control on the patients’ existence, of which the disease has interrupted the normal course. Finally, the quest narrative is characterized by a chronological order, indicating a control of life despite the disease. In particular, the patients underlined that the illness changed their life, providing a new possibility [12].

Then, in order to highlight the relevant themes and sub-themes reported in the collected narratives, according to De Lillo, we performed a qualitative analysis of content (thematic analysis) to assign a text code expressing specific conceptual categories [11]. The thematic analysis followed the six phases identified and descripted by Braun and Clarke [13].

## 3. Results

Ten patients (66.7%) agreed to participate to this study. Among these, 8 narratives were included in the final analysis, while the remaining 2 were excluded as they provided a too-short narrative (56 and 58 words, respectively).

The narratives included have been written by 8 females, with a mean age of 43.3 years (SD 9.9) and a mean disease duration of 13.1 years (SD 7.4). The main demographic features are summarized in Table 1. The analyzed narratives showed a mean length of 436.9 words. At the time of the interview, we found a median SLEDAI-2k value of 1.5 (IQR 2).

As reported in Table 1, six patients provided a quest narrative, one a chaos narrative and the remaining one a restitution narrative. Concerning the quest narrative, the patients described the events by using a chronological order; moreover, they have changed and grown thanks to the challenges imposed by the disease, almost never identified in the early phase. Until the COVID-19 emergency, they were able to control their disease, thanks to regular follow-up and treatments. The narrative classified as chaos described only the period following the COVID-19 pandemic, stressing how this broke an existing balance. Finally, in the restitution narrative, the disease history prevailed, thus the patient focused on their condition as a sick person, searching for healing.

In the text content analysis, we found specific themes, temporally distinct before and after the COVID-19 lockdown, which are summarized in Table 2, accompanied by specific quotations. To summarize, before the COVID-19 emergency, all the patients referred to a good control of disease, despite a troubled past, full of important events (*“My life continued between visits and treatments…I have always been quite well and have had two other wonderful children!”*). Frequently, the narratives described a common sub-theme, related to the long time before the disease diagnosis and to the difficulties encountered in specific life phases (marriage, motherhood, work), overcome with strength and determination, as especially underlined in the quest narrative (*“Finally, the diagnosis, I had been looking for years...”*).

The unexpected arrival of the COVID-19 emergency broke a balance. The patients perceived that they have lost control of their health status, and this caused anxiety and stress, and a lot of fear and concern, for themselves and also for the familiar people around them (*“I don’t know exactly why all this happens, but I am continuously stressed and sad”*). Some patients felt that they *“have no escape”* in case of COVID-19 infection.

The theme of the future was addressed in some narratives. In particular, four patients expressed hope and optimism (*“It will be all right”*)*,* while on the other hand, three believed they had no future due to COVID-19.

Of note, the narratives revealed how patients define their state of health. Some referred to the disease by using positive terms (acceptance), such as “my little wolf” (interviewee 5) or “my lupus” (interviewee 1), while others with a negative connotation, such as interviewee 6, who referred to the disease as “my intruder”.

## 4. Discussion

The present study provided unique insight into the experiences of people with SLE at the time of COVID-19. The narrative approach allowed to find the perspectives of patients in relation to the pandemic, by underlining their concern about the future.

As is widely demonstrated, qualitative research could help to understand the experiences and beliefs about SLE from the patients’ perspective. Data from the literature underlined recurring thematic aspects of living with SLE, in particular uncertainty for the future due to disease and changes in daily functions [14].

The narratives of the patients clearly showed the breaking of a balance induced by the onset of the COVID-19 pandemic, causing fear for their health and for their families. In almost all of the narratives, the disease was well-controlled before the COVID-19 pandemic: the patients underlined that, thanks to the regular follow-ups and the adherence to treatment, their disease was well-controlled, allowing for a normal life. The COVID-19 pandemic, with the consequent lockdown, changed the experiences of SLE patients.

As mentioned above, infections represent one of the most important causes of morbidity in SLE patients, due to the disease itself but also to the immunosuppression related to the treatments [5]. Thus, during the patients’ education, the risk of infections is emphasized, with the need to stop immunosuppressant treatment during an infectious event. The COVID-19 pandemic is a unique situation, determined by a little-known infective agent and for which there is no vaccine. Certainly, this condition could cause stress, anxiety and fear in SLE patients. This impact was well-represented in the narratives of our patients. In particular, the patients perceived a loss of control over their disease and over their health status. The fear was not addressed only for themselves, but also for their families. The COVID-19 pandemic also changed their expectations towards the future: together with optimistic patients, who felt that everything would be fine, there were other patients who could not see a future.

Our study, to the best of our knowledge, evaluated for the first time the perspective of SLE patients with regards to the COVID-19 pandemic. Nonetheless, other studies have recently focused on patients affected by other pathological conditions. The analysis conducted by Forner and colleagues underlined that delays in surgery for cancer patients due to the pandemic resulted in extensive psychosocial distress, and found that patients could mitigate these effects through various coping mechanisms and improved communication with their healthcare teams [15]. Furthermore, the COVID-19 lockdown significantly and negatively impacted younger people with multiple sclerosis (MS), in particular in those with progressive types of MS and psychological symptoms [16]. Nonetheless, as reported by Gleason and colleagues, COVID-19 has disrupted health systems and social services, leading to unprecedented barriers to access and maintain health and addiction services in both inpatient and outpatient settings [17].

## 5. Conclusions

Next to the exclusively scientific perspective of the COVID-19 pandemic, our analysis provides the perspective of SLE patients, highlighting the impact of the pandemic on their lives. Certainly, the small sample size represents the main limitation of our study, together with the lack of a control group, but we aimed at providing the point of view of SLE patients by a narrative approach.

## Figures and Tables

**Table 1:** Socio-demographic information and type of narration for each patient.

Patient ID	Age	Disease Duration (Years)	Education Level	Marital Status	Employment Status	Narration Form
01_CL	44	24	Secondary	Married	Housewife	Quest
02_CM	46	17	Tertiary	Married	Anthropologist	Chaos
03_SF	39	10	Secondary	Married	Bartender	Quest
04_AG	46	12	Secondary	Married	Housewife	Quest
05_SN	34	2	Secondary	Married	Beautician	Quest
06_MP	48	8	Secondary	Married	Employee	Quest
07_PM	61	22	Secondary	Married	Housewife	Quest
08_DG	28	10	Secondary	Married	Shop assistant	Restitution

**Table 2:** Themes and sub-themes identified from patients’ narratives with related quotations.

Themes	Sub-themes	Patients’ ID	Example of Patients’ Quotations
**Pre-COVID-19 Lockdown**	First symptoms and uncertainty	08_DG	I have been affected by Lupus for 10 years, discovered after many researches, after many theories.
	Relief: finally, diagnosis!	05_SN	At first, I felt relief when I learned I had a disease...I wasn’t crazy.
		08_DG	Finally, the diagnosis, I had been looking for years, finally…I was not an imaginary patient, I was not the one who pretended to be ill for not going to school.
	Balance	03_SF	My life continued between visits and treatments. For ten years I have been followed by Dr. XX. Since I have been treated at the Lupus clinic, I have always been quite well and I have had two wonderful children!
	Control	06_MP	Before COVID-19, my illness didn’t scare me because it was under control and I was fine.
**Post-COVID-19 Lockdown**	Fear for herself and her family	05_SN	If I get infected, I don’t know if I WOULD BE SO STRONG... I’m afraid...for me and my family.
		07_PM	Now everything changed. It scares me so much.
	Stress and anxiety	01_CL	I don’t know exactly why all this happens, but I am continuously stressed and sad.
		07_PM	Every little different symptom makes me anxious.
	Vulnerability/loss of control	03_SF	Now this virus comes to upset again the balance obtained over the years.
		04_AG	I have so much but so much fear because if it catches me I don’t think I have a way out.
	I am a fragile person/person at risk	02_CM	I’m worried about being one of those fragile people most exposed to the virus.
		08_DG	We are at risk people.
	I do not have a chance	06_MP	If it catches me, I don’t think I have a way out.
**Future Perspectives**	Hope and optimism	03_SF	But I decided that I don’t want to think about it anymore, because I have always faced my illness.
		01_CL	Come on, let’s stay strong and hard! Do not give up. Everything will be very well.
	“Inexpressible” “No future for me”	02_CM	The future cannot be said, it is not even thinkable yet.
		06_MP	Unlike a few months ago, now I don’t see any future for me.

## Data Availability

The data presented in this study are available on request from the corresponding author. The data are not publicly available.
